# Bioinformatic evidence for a widely distributed, ribosomally produced electron carrier precursor, its maturation proteins, and its nicotinoprotein redox partners

**DOI:** 10.1186/1471-2164-12-21

**Published:** 2011-01-11

**Authors:** Daniel H Haft

**Affiliations:** 1 J. Craig Venter Institute, 9704 Rockville, MD 20850, USA

## Abstract

**Background:**

Enzymes in the radical SAM (rSAM) domain family serve in a wide variety of biological processes, including RNA modification, enzyme activation, bacteriocin core peptide maturation, and cofactor biosynthesis. Evolutionary pressures and relationships to other cellular constituents impose recognizable grammars on each class of rSAM-containing system, shaping patterns in results obtained through various comparative genomics analyses.

**Results:**

An uncharacterized gene cluster found in many Actinobacteria and sporadically in Firmicutes, Chloroflexi, Deltaproteobacteria, and one Archaeal plasmid contains a PqqE-like rSAM protein family that includes Rv0693 from *Mycobacterium tuberculosis*. Members occur clustered with a strikingly well-conserved small polypeptide we designate "mycofactocin," similar in size to bacteriocins and PqqA, precursor of pyrroloquinoline quinone (PQQ). Partial Phylogenetic Profiling (PPP) based on the distribution of these markers identifies the mycofactocin cluster, but also a second tier of high-scoring proteins. This tier, strikingly, is filled with up to thirty-one members per genome from three variant subfamilies that occur, one each, in three unrelated classes of nicotinoproteins. The pattern suggests these variant enzymes require not only NAD(P), but also the novel gene cluster. Further study was conducted using SIMBAL, a PPP-like tool, to search these nicotinoproteins for subsequences best correlated across multiple genomes to the presence of mycofactocin. For both the short chain dehydrogenase/reductase (SDR) and iron-containing dehydrogenase families, aligning SIMBAL's top-scoring sequences to homologous solved crystal structures shows signals centered over NAD(P)-binding sites rather than over substrate-binding or active site residues. Previous studies on some of these proteins have revealed a non-exchangeable NAD cofactor, such that enzymatic activity *in vitro *requires an artificial electron acceptor such as N,N-dimethyl-4-nitrosoaniline (NDMA) for the enzyme to cycle.

**Conclusions:**

Taken together, these findings suggest that the mycofactocin precursor is modified by the Rv0693 family rSAM protein and other enzymes in its cluster. It becomes an electron carrier molecule that serves *in vivo *as NDMA and other artificial electron acceptors do *in vitro*. Subclasses from three different nicotinoprotein families show "only-if" relationships to mycofactocin because they require its presence. This framework suggests a segregated redox pool in which mycofactocin mediates communication among enzymes with non-exchangeable cofactors.

## Background

The number of complete and high-quality draft microbial genomes now exceeds 1000 [1], and provides opportunities to discover and perform initial characterization of novel biological systems by comparative genomics and bioinformatics approaches [2,3]. While systems suggested by conserved operons are readily detected through comparative genomics resources such as STRING [4] and IMG [5], developing meaningful hypotheses for the functions of proteins lacking any characterized homolog and for the discovery and description of novel systems is non-trivial.

However, biological systems completely unrelated to each other in the sense that no component of one shows any homology to any component of the other may resemble each other in their organizing principles. In a *nutrient utilization system*, for example, the set of catabolic enzymes will tend to be accompanied by a transport operon for nutrient uptake. The two sets of genes often will be co-clustered, as well as correlated in their phylogenetic distributions. The system will tend not to be essential, so that one strain may have the system while others lack it, yet distantly related species inhabiting similar environments may share it. These and other commonalities reflect underlying constraints on the relationships among the different parts of a cell, a *biological grammar *that is shared among analogous systems even if they show no common ancestry and no sequence similarities. Patterns learned from one nutrient utilization system can be used to guide the study of another.

In a *bacteriocin system*, a small peptide can undergo multiple forms of processing, including leader peptide removal and various forms of boutique modification that differ from one system to another. The precursor peptide usually but not always is encoded near its maturases. Transporters in the system will be for export (there will be no periplasmic substrate binding protein), and a protease domain may be fused to a permease subunit. Because bacteriocins participate in an evolutionarily rapid arms race of changing toxins and changing defenses, differences in systems even between closely related strains may be profound. Study of the nitrile hydratase-related leader peptide (NHLP) class and nif11 leader peptide (NIF) class of bacteriocin precursor shows a mix-and-match pattern for associating different maturase families interchangeably with target families [6]. Not only can lantibiotic synthases and cyclodehydratases each work on unrelated homology families of precursor peptides, but the same precursor families (conserved in a region that interacts with transporters for cleavage and export) can pair with different, even novel, families of maturases. The biological grammar of bacteriocin systems provides not just a framework for describing known systems, but guidance for the discovery of new systems unrelated to those already known.

Recognizing a biological grammar can help to characterize a system by directing follow-up bioinformatics studies, follow-up experimental work, or both. A particularly important grammar describes *cofactor biosynthesis*. Biological grammars leave telltale signs through the constraints they impose on evolutionary processes. If the cofactor is uncommon and sporadically distributed, as with coenzyme F420 [7] or pyrolloquinoline quinone (PQQ), the vivid evidence for the grammar will show in results from comparative genomics tools such as Partial Phylogenetic Profiling (PPP) [8]. PPP detects correlations between a phylogenetic profile (a listing of genomes) used as a query, and the optimally constructed protein families that could be built around each of the proteins in the genome being queried (see methods). The top tier of results from PPP will consist mostly of the enzymes actually involved in synthesizing the cofactor. In contrast to bacteriocin systems, these enzymes should be consistent from genome to genome. But PPP will also show a second tier enriched in protein families tightly correlated to the presence of the cofactor, usually because they are enzymes directly dependent on that cofactor for their function. Recently, we demonstrated surprising numbers of flavoenzymes, from three paralogous families, nominated by PPP as likely F420-dependent, dozens per genome in *Mycobacterium smegmatis *and other Actinobacteria [7]. A new bioinformatics tool, SIMBAL [9] (see methods), confirmed that it was determinants of cofactor specificity rather than substrate specificity that scored highest. Taylor, et al. [10] have now provided direct experimental confirmation for nine examples of F420-binding enzymes from one of these families.

The present work focuses on Rv0693 and the distinctive subfamily to which it belongs within the radical SAM domain family [11], and on a short peptide with an extremely well conserved C-terminal motif that invariably is encoded in the same gene cluster. Individual subfamilies of radical SAM proteins differ greatly in target and action, and serve as molecular markers for processes as varied as tRNA and rRNA base modification, metalloenzyme maturation, lipid metabolism, and post-translational modification both of proteins to create enzyme active sites, and of short peptides to create bacteriocins and other natural products. Among characterized radical SAM proteins, Rv0693 is most similar to AlbA (Anti-Listerial Bacteriocin A) [12], and to PqqE (Pyrrolo-Quinoline-Quinone biosynthesis E) [13], involved respectively in the biosynthesis of subtilosin A (a bacteriocin) and PQQ (an enzymatic cofactor). A C-x2-C-x5-C-x3-C motif, located C-terminally to the radical SAM domain and involved in binding an additional 4Fe4S cluster [14] is shared exactly by Rv0693 with anaerobic sulfatase maturation proteins, a quinohemoprotein amine dehydrogenase maturation protein, and the Pep1357-cyclizing radical SAM enzyme [15], and shared approximately with AlbA and PqqE. All of these proteins have in common that they perform chemistries on protein or peptide substrates.

It is noteworthy that two profoundly different grammars can describe systems in which a radical SAM enzyme modifies a short peptide. Subtilosin A is an example of a bacteriocin made in this way, while PQQ is an example of an uncommon enzymatic cofactor made from a peptide precursor. The technique of weighing evidence from a variety of comparative genomics approaches against expectations corresponding to different biological grammars, rather than considering homology alone, can provide an opportunity to learn more about novel, uncharacterized systems *in silico *and to provide more concrete and detailed hypotheses of processes and functions.

## Results

### The family of radical SAM protein Rv0693 defines a sporadically distributed conserved gene neighborhood

A further examination of members of the radical SAM domain superfamily [11,16] for member sequences not already described by more specific HMMs in the TIGRFAMs collection [17] revealed a clade that included Rv0693 from *Mycobacterium tuberculosis *H37Rv and its apparent orthologs in numerous other Actinobacteria. A preliminary protein family definition, based on an HMM constructed from a multiple sequence alignment of this putative ortholog set, marked a set of loci and created a phylogenetic profile, from which conserved gene neighborhoods and phylogenetically correlated protein families could be investigated. Partial Phylogenetic Profiling (PPP), a data mining tool that searches for phylogenetically correlated proteins by tuning sets of working definitions of families centered on those proteins for optimal match to the query profile [8], revealed strong matches to several neighboring proteins, including a glycosyltransferase (Rv0696) and a protein of unknown function (Rv0692) with no homologs outside of these neighborhoods. Among the 1450 genomes included in the PPP data set, we determined that the three always occur together, or not at all, both within and outside the Actinobacteria. Non-actinobacterial species included *Thermomicrobium roseum *and *Sphaerobacter thermophilus *from the Chloroflexi, *Pelotomaculum thermopropionicum *and *Desulfotomaculum acetoxidans *from the Firmicutes, and *Geobacter uraniireducens *from the Deltaproteobacteria. Manually refined protein family definitions, with curated seed alignments, HMMs, and curated cutoffs, were constructed and deposited in the TIGRFAMs database [17]. Table 1 lists HMMs created for this study.

**Table 1:** HMMs built during this study

HMM	H37Rv locus	**Len**.	Functional category	Superfamily
TIGR03962	Rv0693	339	radical SAM protein	PF04055

TIGR03965	Rv0696	506	glycosyltransferase, group 2	PF00535

TIGR03967	Rv0692	83	putative mycofactocin-binding	---

TIGR03969	(missed gene call)	23	mycofactocin	---

TIGR03964	Rv0695	239	creatininase family	PF02633

TIGR03966	Rv0694	385	FMN-dependent dehydrogenase	PF01070

TIGR03968	Rv0691c	190	TetR family regulator	PF00440

TIGR03971	Rv0687, Rv2750	281	oxidoreductase, SDR family	PF00106

TIGR03989	Rv0761c, Rv3086	371	oxidoreductase, Zn-containing	PF00107

---	---	---	oxidoreductase, Fe-dependent	PF00465

### Rv0693 belongs to a branch of the radical SAM family in which known or presumed substrates are short peptides

We have compiled a comprehensive list of radical SAM proteins characterized by direct assay, mutational studies, or genome context. The two closest homologs to Rv0693 in the collection AlbA, the lone maturase for subtilosin A biosynthesis [12] and PqqE, part of a cassette of genes required for pyrroquinoline quinone (PQQ) biosynthesis [13,18]. Rv0693 is much more similar to these than to radical SAM proteins of tRNA or rRNA base modification, hydrogenase metallocenter maturation [19], biotin cofactor biosynthesis [20], squalene/hopene biosynthesis[21], sulfatase activation [22], etc. TIGRFAMs model TIGR03963 now defines the Rv0693 family. "PqqE" is the most common misannotation in public databases for members of this family.

### An open reading frame encoding a short peptide with an invariant C-terminus always occurs in the gene neighborhoods defined by the families of Rv0693, Rv0692, and Rv0696

**Figure 1:**
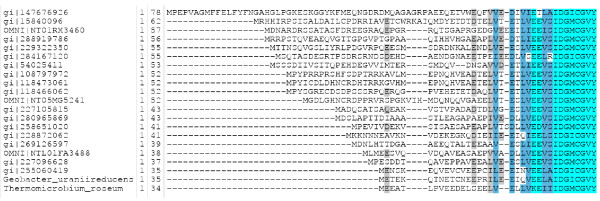
**Multiple sequence alignment of gene predictions for mycofactocin precursors**. All detectable members of the family defined by TIGR03969 were collected, sorted by length, and aligned by MUSCLE [33], and then made non-redundant to 80% sequence identity, preferentially keeping sequences previously treated as genes and available through NCBI. Significant sequence similarity is restricted to the last 23 amino acids; the seven invariant residues occur among the last eight positions.

Inspection of these conserved gene neighborhoods occasionally showed a gene prediction for a short peptide sequence. Multiple alignment of all predicted examples revealed length heterogeneity ranging from 34 to 78 residues, with extreme sequence diversity upstream of a carboxyl-terminal 23-amino acid region of extremely strong sequence conservation, suggesting that the extended, unalignable N-terminal regions largely reflect incorrect start site predictions. Searching six-frame translations of genomes lacking a predicted member of this family revealed that the predicted coding region is universally present. Figure 1 shows a multiple sequence alignment of this predicted protein family, now modeled by TIGR03969. The last four residues, and seven of the last eight, are invariant. Conservation appears to be maintained at the level of protein, not DNA; the last four codons all differ between the genes from *Frankia alni *ACN14a and *Thermomicrobium roseum *DSM5159 while the corresponding amino acids are identical.

The small size (of the conserved region) of this polypeptide is highly suggestive. Such a small polypeptide is unlikely to act as an enzyme or structural protein, but may instead be a substrate. PqqA, the polypeptide precursor of pyrroloquinoline quinone (PQQ) [13], averages 23 amino acids in length, identical to the length of the conserved portion of the alignment in Figure 1, represented in model TIGR03969. Many bacteriocin core peptides are also similar in size, once the leader peptide is removed. Both PqqA and bacteriocins such as subtilosin A are acted upon by radical SAM proteins as part of their peptide maturation [12,18]; the AlbA enzyme introduces three separate linkages from Cys residues main chain atoms of residues elsewhere in the polypeptide chain [12].

A reasonable hypothesis is that the Rv0693 family radical SAM protein modifies the TIGR03969 family peptide to produce a bioactive molecule: a bacteriocin, a redox cofactor like PQQ, or a signaling metabolite such as Pep1357 [15]. The neighboring glycosyltransferase (Rv0696) likely also participates. Based on its near-universal distribution in the genus ***Myco****bacterium *(among many other Actinobacteria), and analogy of its biosynthesis cluster to PQQ **cofact**or and bacteri**ocin **biosynthesis clusters, we suggest the working name "**mycofactocin**" for members of this family.

### The nitrile hydratase/nif11 families expand collections of peptide-modifying radical SAM proteins

Lantibiotic synthases [23] and cyclodehydratases [24] serve as high fidelity markers for bacteriocin maturation clusters, but may be accompanied by additional dehydrogenases, proteases, radical SAM proteins, and other enzymes best recognized as part of the maturation cluster by context. Recently, we showed a mix-and-match relationship between families of precursors and families of maturation enzyme; the nitrile hydratase-related leader peptide (NHLP) and Nif11-related leader peptide (Nif11LP) can associate with a single type of export transporter but with multiple types of modification cluster [6]. Given that AlbA provides an example of a radical SAM enzyme serving as the sole maturase for a bacteriocin, we searched for examples of NHLP and Nif11LP family peptides associated with a radical SAM protein as its only candidate maturase. We found several sets of mutually closely related enzymes, including family TIGR04064 (YP_001959118.1, YP_516403.1, YP_002019717.1, *etc*.), which provide additional examples proteins that are presumed to act on small peptides as substrates, and which possess the C-terminal 4Fe4S-binding motif C-x2-C-x5-C-x3-C that correlates with protein or peptide modification as suggested by Benjdia, *et al*. [14]. These new observations provide additional support that Rv0693 likely targets a small peptide for modification.

### Additional protein families co-cluster with the mycofactocin precursor

The last marker universally found in all mycofactocin gene neighborhoods is the family of Rv0692, now described by TIGR03967. This small protein (about 90 amino acids in size) lacks detectable homology to any protein outside of mycofactocin gene neighborhoods. It might be an enzyme, or subunit thereof, for mycofactocin maturation, but the small size and lack of homology to any known enzyme suggest otherwise. Alternatively, it may be a "scaffolding" protein important during mycofactocin biosynthesis but not after, or a carrier protein, analogous to apocytochrome c, that binds the mature product and mediates its interactions with other proteins. The latter interpretation fits well with the working hypothesis that the mycofactocin system defines a novel redox pool with limited communication with other redox pools such as those of exchangeable NAD or NADP.

Three additional protein families occur, in single copies per genome and co-clustered, in most but not in all species with the mycofactocin gene cluster. TIGR03966 models a protein family that includes Rv0694, described as a flavocytochrome. TIGR03964 models a family of homologs to creatinine amidohydrolase, including Rv0695. TIGR03968 describes a family of putative transcriptional regulators related to TetR. Because these proteins are not universal in mycofactocin species, and not variable in count when they are present, their roles are difficult to infer. The flavocytochrome may be analogous to the NADPH-dependent F420 reductase that enables electron transfer between the F420 and NADPH electron carrier pools.

**Figure 2:**
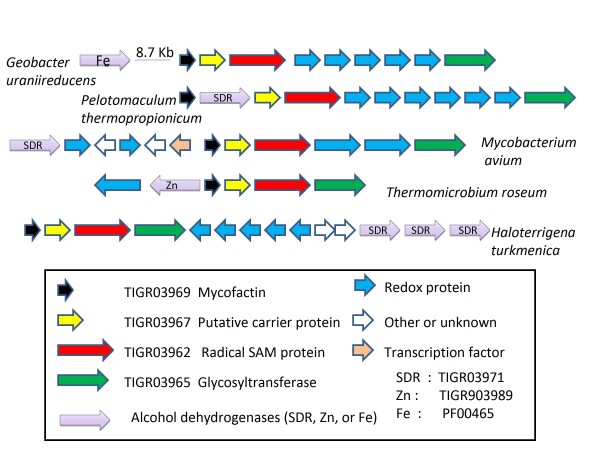
**Mycofactocin gene cluster regions**. Examples of the mycofactocin cluster are shown from *Geobacter uraniireducens *(Deltaproteobacteria), *Pelotomaculum thermopropionicum *(Firmicutes), *Mycobacterium avium *(Actinobacteria), *Thermomicrobium roseum *(Chlorobi), and *Haloterrigena turkmenica *(Archaea). Additional SDR family oxidoreductases for these species, beyond those shown in the mycofactocin cluster, include four Fe-dependent from *G. uraniireducens *and nine from *P. thermopropionicum*, twenty-five SDR and five Zn-dependent from *M. avium*, and four Zn-dependent from *T. roseum*.

The perfect agreement of the radical SAM family TIGR03962, the glycosyltranferase family TIGR03965, and peptide family TIGR03969 creates a reliable phylogenetic profile that enables subsequent data mining to further characterize the systems. The profile is information-rich, not just because of sporadic occurrence outside of the Actinobacteria, but also because of sporadic loss of the system in lineages where it is most prevalent. It occurs in *Frankia alni *ACN14a but not in *Frankia sp*. CcI3, in most *Mycobacterium *species but not in *M. leprae*, in *Geobacter uraniireducens *but not in *Geobacter metallireducens*, *etc*. Exploration beyond the set of 1450 genomes in our all-vs-all comparison set, performed using the non-redundant protein sequence databases at NCBI (April 2010), found that additional species with strong matches to the radical SAM protein and to the glycosyltransferase also contained the putative mycofactocin. These additional species were *Streptomyces sp*. AA4 (an Actinobacterium), *Geobacter sp*. M18 (a Deltaproteobacterium), and *Haloterrigena turkmenica*, an archaeal species in which all components are found on a large plasmid (NC_013744). These additional species were examined for gene content but were not included in data mining analyses. Table 2 shows a list of species contain the mycofactocin gene cluster. Genome regions containing the mycofactocin gene cluster are shown in Figure 2.

**Table 2:** Mycofactocin cluster-containing genomes and linked oxidoreductases

Species/strain name	TIGR03971 (SDR)	TIGR03989(Zn-dep.)	PF00465 (Fe-dep.)
*Desulfotomaculum acetoxidans*	1	0	3

*Frankia alni *ACN14a	10	3	0

*Frankia sp*. EAN1pec	8	0	0

*Geobacter sp*. M18	0	0	5

*Geobacter uraniireducens *Rf4	0	0	6

*Geodermatophilus obscurus *DSM 43160	0	1	1

*Gordonia bronchialis *DSM 43247	1	3	2

*Haloterrigena turkmenica*	5	1	0

*Mycobacterium abscessus*	5	2	0

*Mycobacterium avium *104	26	5	0

*Mycobacterium avium subsp. paratuberculosis *K-10	15	3	0

*Mycobacterium bovis *AF2122/97	2	2	0

*Mycobacterium bovis *BCG str. Pasteur 1173P2	2	2	0

*Mycobacterium bovis *BCG str. Tokyo 172	2	2	0

*Mycobacterium gilvum *PYR-GCK	12	3	0

*Mycobacterium marinum *M	4	5	0

*Mycobacterium smegmatis *str. MC2 155	8	4	0

*Mycobacterium sp. *JLS	15	6	0

*Mycobacterium sp*. KMS	12	5	0

*Mycobacterium sp*. MCS	12	5	0

*Mycobacterium tuberculosis *CDC1551	2	2	0

*Mycobacterium tuberculosis *F11	2	2	0

*Mycobacterium tuberculosis *H37Ra	2	2	0

*Mycobacterium tuberculosis *H37Rv	2	1	0

*Mycobacterium ulcerans *Agy99	3	3	0

*Mycobacterium vanbaalenii *PYR-1	13	3	2

*Nakamurella multipartita *DSM 44233	2	0	2

*Nocardia farcinica *IFM 10152	3	3	2

*Pelotomaculum thermopropionicum *SI	1	0	9

*Rhodococcus erythropolis *PR4	11	5	2

*Rhodococcus erythropolis *SK121	9	4	2

*Rhodococcus jostii *RHA1	16	4	2

*Rhodococcus opacus *B4	15	6	2

*Rubrobacter xylanophilus *DSM 9941	1	1	0

*Saccharopolyspora erythraea *NRRL 2338	0	0	2

*Sphaerobacter thermophilus*	5	1	0

*Streptomyces sp*. AA4	0	1	0

*Thermomicrobium roseum*	0	5	0

*Thermomonospora curvata *DSM 43183	1	1	0

### Partial Phylogenetic Profiling (PPP) based on species distribution of the mycofactocin cluster generates cofactor-like results

As expected, the top tier of scores from PPP belongs to proteins already discussed as invariant markers of the mycofactocin conserved genome neighborhoods. Mycofactocin itself scores poorly because both its small size and its inconsistent gene identification limit its numbers of BLAST hits. Strikingly, additional proteins occur just below the top tier, and for species after species include one or two sets of closely related paralogs from within the same genome. This would be the expected pattern in the case that members of these additional families depend on the system rather than being required as part of it. Such would be the case if the families of paralogs depend on an uncommon molecular cofactor.

Coenzyme PQQ (pyrroloquinoline-quinone) is a broadly but sparsely distributed cofactor biosynthesis system, occurring in about 10% of the 1450 genomes analyzed by systems reconstruction [25]. Table 3 shows PPP results obtained for *Methylobacillus flagellatus *KT, a producer of PQQ. The radical SAM protein PqqE from the PQQ biosynthetic cluster was used as the marker to define the phylogenetic profile. In *M. flagellatus*, the 4^th ^through 8^th ^best hits are paralogs to each other, scattered throughout the genome rather than clustered, and all members of protein family TIGR03075, the PQQ-dependent dehydrogenase, methanol/ethanol family. By contrast, *Mycobacterium smegmatis *carries only one PQQ-dependent alcohol dehydrogenase, MSMEG_3726, readily identified by PPP. This lone member, the top scoring protein not involved in PQQ biosynthesis, lies in the same cassette as the biosynthesis enzymes. These results show a familiar pattern - a single gene dependent on other genes is likely to be clustered with it, while a family of several such genes may be scattered. Note that members of family TIGR03075 score lower by PPP than the PQQ biosynthesis enzymes themselves because their presence in genomes is spottier than that of PQQ itself. Some PQQ producers instead (or in addition) encode members of family TIGR03074, the glucose/quinate/shikimate family of membrane-bound PQQ-dependent dehydrogenases. Even more striking results show certain coenzyme F420-synthesizing Actinobacterial species such as *Mycobacterium smegmatis *to encode large paralogous families of F420-dependent flavoproteins that dominate the PPP results [7]. These two examples, and a recent experimental confirmation [10] for the F420-dependent enzymes, demonstrate that the biological grammar of biosynthetic systems for unusual redox factors can show vividly in PPP results. A second tier of high-scoring proteins, filled with enzymes belonging to expanded paralogous families, suggests that the enzymes depend on that redox factor for their function.

**Table 3:** Partial Phylogenetic Profiling results: PQQ in *Methylobacillus flagellatus *KT

Locus	#Yes	#Total	#Depth	Score	Name
**Mfla_1682**	97	97	134	**-94.521**	**PQQ biosynthesis protein PqqC**

**Mfla_1683**	97	97	136	**-94.521**	**PQQ biosynthesis protein PqqB**

**Mfla_1680**	97	97	135	**-94.521**	**PQQ biosynthesis protein PqqE**

Mfla_2314	73	80	157	**-61.969**	**PQQ-dependent enzyme**

Mfla_1717	84	104	285	**-61.969**	**PQQ-dependent enzyme**

Mfla_1451	84	108	282	**-59.198**	**PQQ-dependent enzyme**

Mfla_2044	77	94	210	**-57.525**	**PQQ-dependent enzyme**

Mfla_0344	79	100	268	**-56.680**	**PQQ-dependent enzyme**

**Mfla_1681**	53	53	83	**-51.646**	**PQQ biosynthesis protein PqqD**

Mfla_2043	42	43	69	-39.341	extracellular solute-binding protein

Mfla_2313	42	43	59	-39.341	extracellular solute-binding protein

PPP for *Mycobacterium avium*, using the mycofactocin radical SAM protein model to generate the query profile, shows an extraordinary number of oxidoreductases of the short chain dehydrogenase/reductase family (SDR) described by PF00106, twenty-six of the top fifty hits. An additional five of the top fifty hits belong to PF00107, oxidoreductases of the zinc-binding dehydrogenase family. These two cohorts of paralogous proteins each belong to a distinctive clade within its parent family. TIGR03971 represents that clade within family PF00106, and TIGR03989 represents the clade within PF00107. Each of these newly defined clades is exclusive to species with the mycofactocin/radical SAM/glycosyltransferase system. The pattern repeats in *Rhodococcus jostii *RHA1, with PPP results shown in Table 4, where the top 26 hits all belong to the mycofactocin operon, to family TIGR03971 (sixteen members), or to family TIGR03989 (four members). In both *Geobacter uraniireducens *and *Desulfotomaculum acetoxidans*, a member of the iron-dependent alcohol dehydrogenase family described by PF00465 scores high by PPP and occurs next to the radical SAM protein. The clustered member in *G. uraniireducens *is one of three closely related paralogs from family PF00465 that all score among the top 1% of proteins by PPP.

**Table 4:** PPP results: Mycofactocin system in *Rhodococcus jostii *RHA1

gi #	#Yes	#Total	#Depth	Score	Family	HMM
**111023035**	28	28	37	-42.079	**rSAM**	TIGR03962

**111023041**	27	28	39	-39.142	**glycosyltransferase**	TIGR03965

111017396	24	24	204	-36.067	**SDR**	TIGR03971

**111023038**	24	25	188	-34.683	**SDR**	TIGR03971

111022782	24	25	172	-34.683	**SDR **(truncated)	TIGR03971

111025759	24	25	219	-34.683	**SDR**	TIGR03971

**111023036**	24	25	33	-34.683	**heme/flavin DH**	TIGR03966

111017413	24	25	125	-34.683	**SDR**	TIGR03971

111026996	24	25	189	-34.683	**SDR**	TIGR03971

111019823	23	23	88	-34.564	**Zn**	TIGR03989

111019488	23	23	80	-34.564	**Zn**	TIGR03989

111018217	23	23	83	-34.564	**Zn**	TIGR03989

**111023034**	23	23	32	-34.564	**RPExFGAL protein**	TIGR03967

111026997	23	23	69	-34.564	**Zn**	TIGR03989

111026295	23	23	68	-34.564	**SDR**	TIGR03971

111019471	23	23	191	-34.564	**SDR**	TIGR03971

111020800	24	26	188	-33.582	**SDR**	TIGR03971

111024993	24	26	205	-33.582	**SDR**	TIGR03971

111025414	24	26	225	-33.582	**SDR**	TIGR03971

111025862	23	24	110	-33.198	**SDR**	TIGR03971

**111023033**	23	24	33	-33.198	**TetR regulator**	TIGR03968

111024115	22	22	152	-33.062	**SDR**	TIGR03971

111019472	22	22	139	-33.062	**SDR**	TIGR03971

111026871	22	22	131	-33.062	**SDR**	TIGR03971

**111023040**	22	22	29	-33.062	**creatininase**	TIGR03964

111022752	21	21	84	-31.559	**SDR**	TIGR03971

111023342	22	26	33	-28.940	NADH pyrophosphatase	

111018894	20	21	52	-28.747	triacylglycerol lipase	

### SIMBAL analysis of the SDR family highlights cofactor-binding rather than substrate-binding residues

Multiple members of PF00106, the short chain alcohol dehydrogenase (SDR) family of oxidoreductases, occur in the top 1% of PPP results for various mycofactocin-encoding genomes. In fact, all of these SDR proteins belong to a distinctive clade within the PF00106 family, now described by TIGRFAMs model TIGR03971. This wing of the SDR family has undergone a significant paralogous family expansion in multiple members of the Actinobacteria, with two members (Rv0687 and Rv2750) in *Mycobacterium tuberculosis*, nine in *Rhodococcus erythropolis *SK121, and twenty-six in *M. avium*. In this family, a stereoselective carveol dehydrogenase from *Rhodococcus erythropolis *DCL14 has been characterized as a novel nicotinoprotein in the SDR family, with sequence alignment suggesting an extra loop in the NAD-binding region [26]; the presence of this loop is conserved across members of TIGR03971. Experimental characterization of that protein revealed that the NAD(H) cofactor, although not covalently bound, is not exchangeable. Demonstrating enzyme activity in vitro required use of an artificial electron acceptor, dichlorophenolindophenol (DCPIP), to reduce the NAD cofactor *in situ*. No member of the TIGR03971 family has a solved crystal structure, but structure 1NFQ is available from PDB as a ternary complex with bound NAD(+) and androsterone for Rv2002 [27], which is one of the most closely related members of the SDR family outside of the TIGR03971 family. This NADH-dependent 3alpha, 20beta-hydroxysteroid dehydrogenase is described as having a catalytic triad Ser-140,Tyr-153, and Lys-157, with residue Asp-38 called critical for cofactor specificity. These four critical residues remain invariant in TIGR03971 sequences.

**Figure 3:**
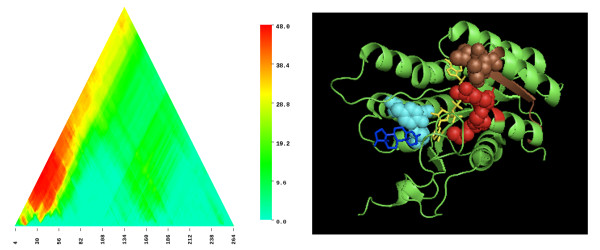
**SDR family dehydrogenase SIMBAL heat map and related structure**. **Panel A**: SIMBAL map for 267-residue protein PTH_0592 from *Pelotomaculum thermopropionicum *SI. Data sets numbered 974 sequences in the TRUE partition and 6998 in the FALSE partition after being made non-redundant to less than 80% sequence identity. The X coordinate represents the location of the center of each subsequence along the full-length protein sequence, the Y coordinate represents the size of subsequence tested. The plot is triangular, narrowing as it rises, because longer subsequences are more constrained and have less room to slide from N-terminus to C-terminus. **Panel B**: Structure 1NFQ of Rv2002, an NADH-dependent 3alpha, 20beta-hydroxysteroid dehydrogenase in the SDR family. Bound NAD+ is shown in yellow and androsterone in blue. Active site resides Ser-140, Tyr-153, and Lys-157 are shown in cyan with their side chains as space-filling spheres. Highlighted in brown and red are regions from Rv2002 that map by pairwise alignment to the locally top SIMBAL hit sequences from PTH_0592 at subsequence sizes of 8 (brown) and size 12 (red), both taken from within the absolute highest scoring subsequence.

A limitation of PPP [8] is that it performs its analyses on full-length proteins. Recently, we addressed this limitation by introducing SIMBAL, Sites Inferred by Metabolic Background Assertion Labeling [9], which can find key domains or motifs within a protein through a mechanism similar to PPP (see methods). SIMBAL heat maps generated for members of TIGR03971 always show pronounced peaks centered within the N-terminal 60 residues. For sequences outside of the Actinobacteria, these peaks dramatically outscore the apex of the triangular heat map, the single point that represents the full length of the protein. The sequence represented in the heat map in Figure 3 is for a member of the TIGR03971 subfamily of the SDR superfamily from *Pelotomaculum thermopropionicum*, a member of the Firmicutes. The absolute peak score occurs for substrings drawn from within the N-terminal 60 residues; 56 of the 58 most closely related homologous subsequences as scored by BLAST come from genomes that also encode the mycofactocin system. Comparable length subsequences from elsewhere in the sequence score much more poorly, reflected by colder blue and green colors in the heat map. PPP had already called attention to the "only-if" relationship between mycofactocin and the TIGR03971 family of carveol dehydrogenase-like SDR, with its altered NAD binding loop and non-exchangeable NAD cofactor. The SIMBAL result, in which a local hotspot outscores the full-length sequence, suggests that PPP may actually be understating the strength of the linkage between the N-terminal region and the mycofactocin system.

The second panel in Figure 3 shows the crystal structure 1NFQ, a member of the SDR family with bound NAD and substrate [27]. No SDR enzyme within family TIGR03971 has been crystallized, but aligning sequences found by SIMBAL to the structure of INFQ can be informative. Although SIMBAL found a global peak at in the N-terminal 60 residues using a long subsequence, examining results for shorter subsequences from this stretch shows the global peak resolving into two smaller local peaks. The two local peaks both represent residues that bind the NAD cofactor directly, while sitting far from the position of the substrate-binding pocket and active site residues.

### The zinc-dependent alcohol dehydrogenase family also contains a subfamily restricted to mycofactocin-containing species

Performing PPP against multiple genomes, with the set of mycofactocin-encoding species as the query profile each time, finds several species in which multiple proteins from the zinc dependent alcohol dehydrogenase family of PF00107 score among the top-scoring proteins. *Mycobacterium avium *contains 5 in the top 50. Collecting several of these high-scoring proteins yielded a seed alignment from which model TIGR03989 was constructed and added to the TIGRFAMs database. Strong support that the connection to the mycofactocin profile is meaningful rather than fortuitous comes from the occurrence of TIGR03989 members in or near the mycofactocin cluster in genomes such as *Thermomicrobium roseum *and *Haloterrigena turkmenica *plasmid pHTUR01 [see Figure 2]. As with the SDR subfamily in TIGR03971, occurrence is not universal among mycofactocin producers, but rather can include multiple members per genome. Again, the structure of this relationship suggests a family of enzymes with a shared dependency on a necessary cofactor.

**Figure 4:**
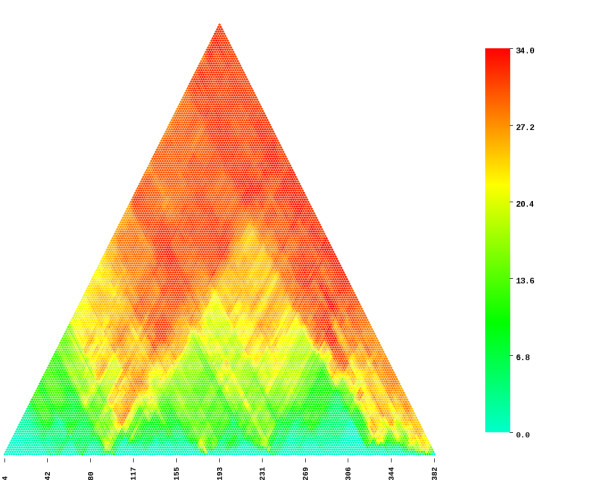
**The SIMBAL heat map for gi|284167095 from *Haloterrigena turkmenica*, a Zn-dependent protein from family TIGR03989**. SIMBAL training sets contained 590 sequences in the TRUE partition and 3620 in the FALSE partition after making the sets non-redundant to no more than 80% sequence identity. The apex score of 30.1 nears that of highest local score, consistent with the ability of TIGR03989 to identify large numbers of members found exclusively in mycofactocin-producing species. Interesting features include extended cool (blue and green) regions such as the N-terminal region of about 80 residues, which contains regions well conserved among Zn-dependent alcohol dehydrogenases yet apparently poorly predictive for whether or not a matching protein's genome of origin contains the mycofactocin cluster.

The zinc-dependent alcohol dehydrogenase family modeled by PF00107 contains examples of enzymes that require, besides NAD(P), a second cofactor. These include the mycothiol-dependent formaldehyde dehydrogenase from *Mycobacterium smegmatis *and a glutathione-dependent formaldehyde dehydrogenase from *Homo sapiens *- two homologous enzymes that both bind NAD, and both act on adducts of the same substrate, but that rely on different coenzymes [28]. SIMBAL might have been expected to show clearly a mycofactocin interaction site. For several different members of family TIGR03989, however, probing with SIMBAL reveals that the best scores from individual subsequences are merely comparable to the score for the full-length sequence, rather than clearly exceeding it. This flatness limits prospects for producing strong inference from SIMBAL heat maps. The heat map for member gi|28416709 from *Haloterrigena *is shown for completeness in Figure 4. It does not single out any particular site or sites as being the most critical for recognizing that a zinc-dependent dehydrogenase originates from a genome that encodes the mycofactocin systems. It does reveal, however, that best matches to certain regions of protein structure, such as those associated with homodimerization rather than with cofactor binding, show very little correlation to the mycofactocin system.

### The iron-containing alcohol dehydrogenase family shows peak scores at the NAD binding site

A third protein family in which several paralogs from the same genome occur among sequences with top scores by PPP is again a set of nicotinoproteins, this time the iron-activated alcohol dehydrogenase family modeled by Pfam HMM PF00465. Once again, the link is supported by gene location, close to the mycofactocin biosynthesis cluster. And again, the subfamily formed by PPP-nominated members of the larger family, when tested, is found to be absent outside of mycofactocin producers, and sporadic rather than universal among them. This relationship once again supports the notion that the dehydrogenase subfamily has a dependency on mycofactocin, rather than a role in producing it.

**Figure 5:**
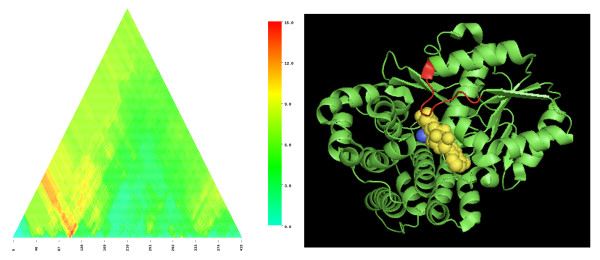
**Iron-dependent dehydrogenase SIMBAL heat map and related structure**. **Panel A **shows the SIMBAL heat map for Dtox_4270, a 419-residue group III iron-dependent dehydrogenase from *Desulfotomaculum acetoxidans *DSM 771. The training set contains 62 sequences in the TRUE partition and 1555 in the FALSE partition. This heat map shows a plume rising from a short subsequence TSNPKDYE, homologous to the sequence VPNPTITV in the sequence of lactaldehyde:1,2-propanediol oxidoreductase Of *Escherichia Coli*, which has a solved crystal structure 2BL4. **Panel B **shows the secondary structure cartoon of 2BL4 in green. The NAD cofactor is shown as space-filling spheres in yellow, and the iron atom as a blue sphere. The sequence identified by homology to the SIMBAL hot spot from panel A is shown in red, making close contact with the NAD but not with the iron atom. The location of the hot spot is consistent with a role in controlling the ability of NAD to exchange electrons to other redox carriers.

SIMBAL plots based on two members from outside the Actinobacteria, Gura_3568 from *Geobacter uraniireducens *Rf4 (Deltaproteobacteria) and Dtox_4270 from *Desulfotomaculum acdetoxidans *DSM 771 (Clostridia), give very similar heat maps. Figure 5 shows the SIMBAL head map for Dtox_4270, a member of the iron-activated dehydrogenase family 419 residues in length. The heat map shows a plume centered on the sequence TSNPKDYEVH. Pairwise alignment identifies a corresponding sequence in the crystal structure 2BL4 of FucO, a member from Escherichia coli of this group of "iron-activated" hydrogenases. This sequence sits over the NAD, making direct contact with the cofactor while sitting far from the active site region iron atom. A loop in this position easily could regulate the ability of the nicotinoprotein to give access by other components of electron transport chains to its bound NAD. The interpretation is guarded, however, as the total number of sequences in the YES partition is relatively small. Furthermore, it has not been possible to build an HMM that finds all PPP-nominated members of this family while systematically excluding all homologs from mycofactocin-negative genomes. It may be that recruitment of PF00465 family iron-dependent dehydrogenases to the hypothesized mycofactocin system has occurred two or more times independently.

All three nicotinoprotein families identified by PPP are supported by co-clustering with the mycofactocin gene cluster in multiple species. The subfamily with the largest numbers of paralogs identified by PPP vs. the mycofactocin cluster is the TIGR03971 branch of the SDR family. Certain members of TIGR03971 have a literature describing an unusual non-exchangeable NAD binding site and unknown physiological electron acceptor. SIMBAL analysis shows for the TIGR03971 family, the salient feature in the heat map is exactly the NAD-binding region. The finding is repeated in the much smaller set of iron-dependent oxidoreductases, with SIMBAL analysis pointing precisely to the NAD. The fact that several of these oxidoreductases have been at least partially characterized, and shown to contain NAD *per se*, leads us to hypothesize that mycofactocin, perhaps held by a carrier protein, interacts with these proteins near the site of its tightly bound NAD in order to allow electron transport, rather than serving as an NAD analog able to bind in place of NAD.

## Discussion

Results from PPP suggest that several paralogous families of enzymes, small clades of nicotinoproteins within each of the SDR, Zn-dependent, and Fe-dependent alcohol dehydrogenase families, are functionally linked to an operon that contains a probable polypeptide-modifying radical SAM protein and its apparent target. The presence of this operon seems not only to allow the occurrence of members of these nicotinoprotein special subfamilies within genomes, but provide opportunities for considerable expansion of those subfamilies, as seen by the twenty-six SDR in *Mycobacterium avium*.

In some Rv0693 family-containing Actinobacteria, there have been observations of oxidoreductases whose NAD cofactor is not exchangeable, whose activity therefore can be observed only in the presence of artificial electron acceptors, and whose electron transfer partners *in vivo *are unknown. These nicotinoproteins include carveol dehydrogenase from *Rhodococcus erythropolis *DCL14[26] and several alcohol and aldehyde dehydrogenases from *Amycolatopsis methanolica *[29,30]. They belong to three mutually non-homologous alcohol dehydrogenase families: the Short Chain Dehydrogenase/Reductase family (SDR) of Pfam model [16] PF00106, the zinc-binding family of PF00107, and the iron-activated family of PF00465. But further, they belong specifically to the particular branches of these families detected by PPP when the species distribution of the mycofactocin cluster is used as the query profile.

Each of the three subfamilies of interest may be absent from any one mycofactocin-containing genome, but more commonly occurs with multiple members present. On the other hand, the mycofactocin system never occurs unless at least one of the three subfamilies has members present. When the mycofactocin operon is absent in a close taxonomic neighbor (as in *M. leprae*, *Frankia sp. *CcI3, *Geobacter metallireducens*, etc.) to a species that has the operon, then these three subfamilies of nicotinoproteins are absent as well. This pattern suggests that each of these nicotinoprotein subfamilies depends on the presence of the mycofactocin system, while the utility of the mycofactocin system to a species depends on the presence of at least one of the three subfamilies. Overall, these relationships suggest nicotinoproteins that are linked to mycofactocin through cofactor interactions rather than, say, through pools of substrates.

Mycofactocin is not associated with any transporters or any recognizable immunity proteins. The precursor lacks an apparent signal peptide or cleavage signal. The final four residues of mycofactocin, Cys-Gly-Val-Tyr, are invariant from Actinobacteria to Firmicutes to Proteobacteria to Archaea. Tyrosine is aromatic, consistent with an electron carrier role. Cysteine is a commonly modified residue in bacteriocin maturation, as in the three Cys side chains bridged to other main chain atoms of other residues by a AlbA during subtilosin A biosynthesis. An adjacent glycine allows for the minimum possible steric hindrance to a possible chemical rearrangement of the peptide backbone. Further study of this system will require direct experimental efforts to purify and characterize mycofactocin from natural or reconstituted systems, and to screen more members of the oxidoreductase families for activity in the presence of artificial electron acceptors. Meanwhile, this study suggests that additional radical SAM enzymes, and perhaps other peptide modification enzymes known from the study of bacteriocin biosynthesis systems, may act upon additional classes of short peptides to create products built not for export, but rather for direct roles in cellular metabolism.

## Conclusion

A broadly but sparsely distributed conserved operon, found in Gram-positive and Gram-negative bacteria and in the Archaea, connects a novel radical SAM protein family to a short peptide, nearly invariant at its C-terminus, that may be its substrate. The putative product, called mycofactocin, occurs only in genomes that encode variant forms of NAD-dependent dehydrogenases in which the NAD cofactors, when examined, were not exchangeable. Investigation of these dehydrogenases shows that sequence similarities near the cofactor binding site offer the best prediction as to whether or not a related dehydrogenase is from a mycofactocin-encoding genome. The results suggest the existence of a novel natural product with electron carrier activity, partnered with novel classes of redox enzymes, in *Mycobacterium tuberculosis *and numerous other bacteria.

## Methods

### Partial Phylogenetic Profiling

Obtaining a list of best BLAST [31] hits to a protein and picking some arbitrary cutoff to truncate the list provides a crude working description for a protein family. Certain well-chosen cutoffs can do particularly well both including proteins that share a specific function and excluding those that don't, but such cutoffs may be hard to determine. However, given a phylogenetic profile to serve as a reference, and evaluating all possible BLAST score cutoffs to find where the list of genomes of origin for the included set best matches the phylogenetic profile, makes it possible to choose candidate cutoffs correctly. Once each protein in a genome receives an individually optimized cutoff to find how well it can match the profile, all proteins can be ranked. Partial Phylogenetic Profiling (PPP) [8,32] produces this ranked list, simultaneously nominating the best scoring proteins additional components of multigene systems and suggesting reasonable preliminary cutoffs for new protein family definitions.

Partial Phylogenetic Profiling (PPP) [8] was performed using pre-computed BLAST results on 1450 complete or high quality draft prokaryotic genomes. PPP explores a genome for proteins that may belong to families closely tied to some taxonomic distribution (usually defined by the presence of some other marker). To perform its search, it scores each protein independently, running down its list of most closely matching proteins by BLAST and selecting the cutoff for which the list of species encountered most closely matches the profile used to conduct the query.

TIGRFAMs model TIGR03962 was used as the protein family decision rule, or marker, to determine which genomes contain the mycofactocin system, thus generating the query profile. This species list was verified by searches with the glycosyltransferase model TIGR03965 and the possible carrier protein model TIGR03967. For additional study, genomes not included in the set of 1450 were searched at NCBI using PSI-BLAST [31] with manual review at each iterative step.

### HMM construction

Hidden Markov Model construction follows manual review of multiple sequence alignment membership and alignment quality, inferred molecular phylogenetic trees, and cutoff scores, according to practices described for the TIGRFAMs project [17]. Multiple sequence alignments were generated with MUSCLE [33].

### SIMBAL

SIMBAL, or Sites Inferred by Metabolic Assertion Labeling, functions much like a variant of PPP that can generate independently calculated scores for any subsequence of any length. For a single protein family (e.g. PF00106), members found in the collection of complete reference genomes are split into TRUE and FALSE partitions of a training set, based not on any known attribute of the protein itself, but rather on a computed attribute such as whether or not its genome of origin appears capable of a particular biosynthesis. Next, one member sequence is selected to test, and all possible subsequences are compared by BLAST to a combined data set containing both partitions. Subsequences score best when sequences from the TRUE partition dominate the BLAST results; for each subsequence, exploration of different BLAST score cutoffs finds the cutoff that gives the best possible score for the subsequence.

SIMBAL scores can be shown graphically in the form of a heat map, with the x-axis representing the center of each subsequence, the y-axis representing the length of the subsequence, and points colored on a scale from least significant (blue) to most signifant (red). Because there are fewer ways to select longer subsequences, culminating in just one way to select the full-length sequence, SIMBAL heat maps are triangular, with the apex representing the whole protein. The score at the apex, therefore, most nearly resembles Partial Phylogenetic Profiling, while plumes in the heat map that rise from short subsequences indicate sites that contribute strongly both to specific function and to PPP score. Aligning these high-scoring subsequences to sequences with solved three-dimensional structures can distinguish whether SIMBAL peaks correspond to sites that interact with substrates, with cofactors, or with other proteins.

Training sets for SIMBAL [9] were performed with by using the phylogenetic profiles to guide the partitioning of protein families PF00106, PF00107, and PF0465 [16]. Fragmentary sequences were removed, and data sets were thinned by removing one of every pair of sequences greater than 80% identical in sequence. Training set sizes were 974 TRUE and 6998 FALSE (12.2% TRUE) for the SDR family PF00106, 590 TRUE and 3620 FALSE from Zn-dependent family PF00107 (14% TRUE), and 62 TRUE, 1555 FALSE from the Fe-dependent family PF00465) (3.8% TRUE). Crystal structure interpretation was performed with the aid of MacPyMol http://www.pymol.org/, using crystal structures downloaded from PDB. SIMBAL hot spot sequences were aligned to their own full-length sequences and to the corresponding sequences of proteins with solved crystal structures, and the alignments were inspected manually to confirm accuracy.

## List of abbreviations used

HMM: hidden Markov model; NAD: nicotine adenine dinucleotide; NDMA: N,N-dimethyl-4-nitrosoaniline; PPP: Partial Phylogenetic Profiling; PQQ: pyrroloquinoline-quinone; SIMBAL: Sites Inferred by Metabolic Background Assertion Labelling; SDR: short chain dehydrogenase/reductase

## Authors' contributions

DHH, as sole author, performed all protein family construction, data analysis and interpretation, and manuscript preparation.

## Author's information

DHH is an Assistant Professor at the J. Craig Venter Institute, who works on developing databases, algorithms, and approaches that can drive more effective genome annotation.
